# VirSim - a Model to Support Pandemic Policy Making

**DOI:** 10.1371/currents.RRN1181

**Published:** 2010-09-22

**Authors:** Tobias Fasth, Marcus Ihlar, Lisa Brouwers

**Affiliations:** ^*^Stockholm University, Department of Computer and Systems Sciences,; ^†^Stockholm University, Department of Computer and Systems Sciences and ^‡^Swedish Institute for Infectious Disease Control, Department of Epidemiology, Solna, Sweden

## Abstract

A simulation model called VirSim was developed to aid policy making in Sweden. The model simulates the spread of pandemic influenza, using real population data on a national and regional level. Swedish authorities wanted a model that was both quick to run and to implement as a complement to the existing model MicroSim. The possibility to interactively investigate the effect of varying different assumptions was an important aspect. The VirSim model proved useful for comparing different interventions strategies, and for forecasting the societal burden in terms of hospitalization and workplace absenteeism. This paper points out the usefulness of System Dynamics models in public policy making, as a complement to more detailed and time-consuming models.

## Introduction

During the planning for the outbreak of pandemic A(H1N1) influenza in Sweden, the National Board of Health and Welfare initiated a research for the development of a decision support tool to complement the individual based, total population model MicroSim [1]. The primary requirements for this tool was that it should support scenario analysis, i.e. to run “what – if” experiments. It was also important that the tool should be implemented in a short time frame and easy to learn and run.  The quality of the model output was also important, but at this stage exact prognoses were not essential – the task for this tool was to produce acceptable forecasts in a timely manner. 

In epidemiology, so-called SIR, or SEIR, models are very common to represent the spread of disease in a population. People are divided into three (or four) compartments; susceptible (S), exposed (E), infected (I) and recovered (R). A system of coupled differential equations governs the flows between the different compartments over time, people becoming infected move from S to I and people who recover (or die) move from I to R. System Dynamics is a natural choice for implementing models simulating transmission processes, since the methodology presupposes a holistic approach and focuses on how the parts in the system affect each other with reinforcing or balancing feedback loops [2] [3].

The model, called, VirSim was developed with the following main goals in mind:

- to create a SEIR model based on available population data and disease specific knowledge, including contact rates
- to extend the model with a Graphical User Interface (GUI) for easy configuration of simulation parameters

## Method

The model, called VirSim, was developed in the modelling environment AnyLogic.Three identical SEIR models were created, each one representing a different age group, to estimate the spread of infection between and within age-groups.  Intervention measures were added to the model and a graphical user interface was created for displaying results and for allowing the user to interactively set the parameters before each simulation run (Figure 1 , 2 and 3). To enable regional analysis, population data from each county in Sweden was added as well as an option in the GUI for region selection. A typical simulation run takes approximately one minute to run on a standard PC.

**Figure:**
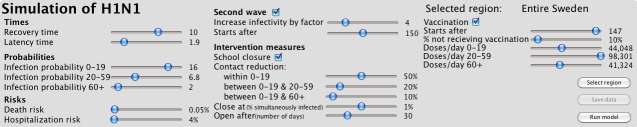

Figure 1: Snapshot of the interface that allows users to change parameter values between runs.   

**Figure:**
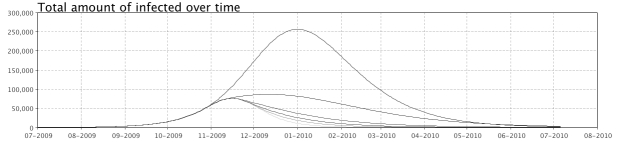

Figure 2: Graphs displaying results from previous simulation runs are shown at the lower half of the view displayed between runs.  

**Figure:**
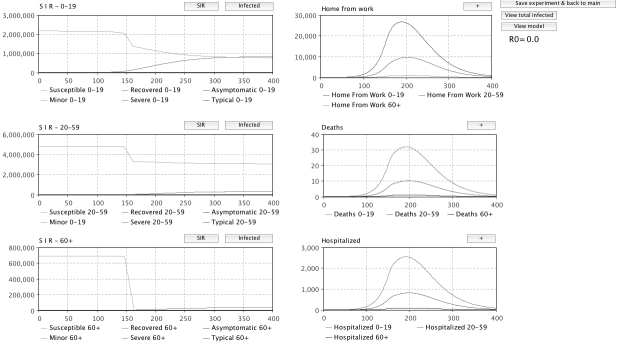

Figure 3: Snapshot of a runtime view. Three SIR-graphs are displayed, one for each population as well as graphs displaying the amount of people home from work, the number of deaths and number of hospitalizations over time. Each graph can be expanded so that it occupies the entire screen.

### Model description

The Swedish population is divided into three age groups (0-19, 20-59 and 60+), data is obtained from Statistics Sweden [4]. Infection is spread through contacts between susceptible and infected individuals. Contacts within and between age groups are based on age specific contact data (see Table 1), obtained from empirical studies in which persons kept diaries of the number of social contacts per day [5].     

**Table:** 

Age group	0-19	20-59	60+
0-19	43.45	27.72	5.42
20-59	27.72	16.84	8.30
60+	5.42	8.30	7.75

Table 1: Age-specific contact data  

Infection is followed by a latent period, which is assumed to last 1.9 days [6] [7]. No symptoms show during this period. When symptoms occur, people may become severely ill or only mildly. This variability is captured by dividing the group of infected people into four classes with different disease severity profiles, ranging from asymptomatic (no symptoms) to severe. The disease profile distribution is the same for all age groups and is based on the distribution used in the MicroSim model [8]:

- Asymptomatic 16%
- Mild 34%
- Typical 40%
- Severe 10%

 


Each disease profile is further associated with a number of sick leave days. This affects the societal costs due to absenteeism, but also the disease spread since the number of daily contacts is reduced when staying home from work.  The following assumptions are made:

- Asymptomatic: home from work 0 days
- Mild: home from work 1 day
- Typical: home from work 3 days
- Severe: home from work 8 days

 


The time to recover from an infection is assumed to be 10 days from infection. When recovered people become immune and are no longer infectious. No prior immunity to the disease is assumed, which is the typical case for a pandemic when the virus has mutated and is unknown to our immune systems. However, the model allows the user to interactively set the infection risk for different age groups. This makes it possible to change the assumption when better data becomes available. It seems like old people are less likely to be infected, it is not sure if this is a result of some level or immunity or if it is an effect of fewer social contacts. 

### Policy experiments

To illustrate how the VirSim model can be used in a structured way to investigate the effect of different policy strategies under different conditions, a series of experiments was conducted.  Here, two different policies were tested; vaccination and school closure. Different assumptions are important for how efficient the policies are: for vaccination the uptake rate, or coverage, is pivotal, and for school closure the length of the closure are very important. The experiments are designed to show how sensitive the policies are to these assumptions.  A number of underlying assumptions are also relevant for the outcome; these were not varied in the experiments: 

- The simulation starts the 1st of June 2009 with 50 initially infected individuals divided between the age groups
- The base-line scenario is calibrated to generate a similar age distribution of infected people as reported in New Zealand [9]

- 0-19=64%
- 20-59=32%
- 60+=4%

- The base-line scenario is calibrated to generate a relatively mild outbreak (R0 1.2)

 


In the first experiments, investigating vaccination, following values were used: 

- The start time of the vaccination is set to the 25th of October 2009, (based on the Swedish vaccination program [10])
- One vaccine dose will be administered to each individual, except for people aged 6 months to 13 years who will receive two doses^.^
- The vaccination doses will be administered at such a pace that it takes 7 weeks to vaccinate the entire population with one dose
- Vaccine effectiveness:

- One dose : 80%  become fully immune, but for people aged 60+ 100% become fully immune
- Two doses: 85% become fully immune

 


Five different vaccination experiments were performed, where the coverage was varied. 30%, 50%, 60%, 70% and 90% of the population were vaccinated in the different runs.  

In the second series of experiments, focusing on school closure, following values were used:

- Initialization: the schools close when 1% of population 0-19 are infected.
- A school closure leads to a reduction of contacts within the population, the following reductions within and between age-groups were assumed:

- Contacts within population 0-19 are reduced with 50%
- Contacts between population 0-19 and 20-59 are reduced with 20%
- Contacts between population 0-19 and 60 plus are reduced with 10%

 


Three school closure experiments were run; where the closure times were 7 days, 21 days and 28 days.  

The third series of experiment investigated the effect of combining the two policies; 60% vaccination coverage was combined with a 21-day school closure using the same assumptions as stated above.

## Results

The vaccination experiments show that a higher coverage yields fewer infections, the peak is also delayed when the coverage increases.  Figure 4 shows that the peak of new infections in the base-run is reached after 29 weeks. With vaccination coverage of 30 % the outbreak peaks three weeks earlier, and for the 90%, 70%, 60% and 50%-scenarios the peak is six weeks earlier. In Table 2 it is shown that the number of infections is 54% lower with vaccine coverage of 30% compared to the base line run with no vaccination. The remaining scenarios show a decrease between 74% and 82%, the higher the coverage the larger the decrease.   

**Figure:**
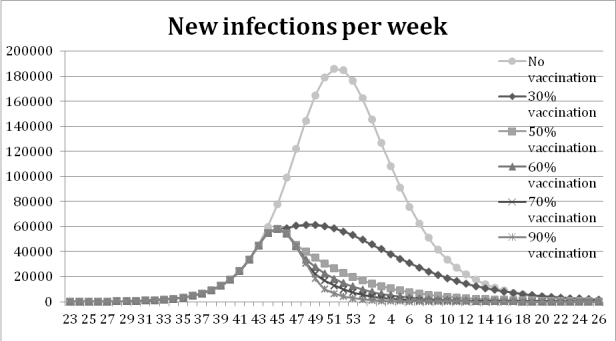

Figure 4: Results from the vaccination experiment measured in new infections per week. The peak of new infections is reached at week 51 in the base run, at week 48 with a coverage rate of 30% and at week 45 with coverage rates between 50% and 90%.  

**Table:** 

Vaccination coverage	Infected	Decrease
No vaccination	2.588.164	0.0%
30%	1.191.654	54%
50%	664.212	74%
60%	555.514	79%
70%	499.489	81%
90%	454.378	82%

Table 2: Results from the experiment with vaccination, measured in the decrease of the total number of infected compared to the base run.  

The school closure experiments show that a one-week school closure delays the peak of infection one week compared to the base-run. With a three- and four-week school closure the peak is reached three and four weeks later than in the base-run, see Figure 5. All scenarios with school closure display a decrease in the total number of infections, Table 3.   

**Figure:**
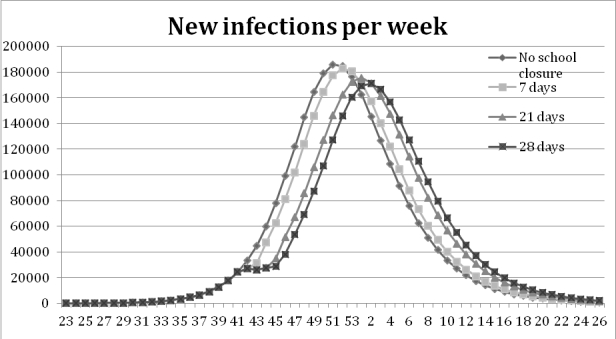

Figure 5: Results from the school closure experiments measured in new infections per week. The graphs show that the peak is delayed when the school stays closed a longer period, four weeks at most.   

**Table:** 

Duration of closure	Infected	Decrease
No closure (base run)	2.588.164	0.0%
7 days	2.579.317	0.3%
21 days	2.561.902	1.0%
28 days	2.553.585	1.3%

Table 3: Results from the experiment with school closure, measured in reduction of infections compared to the base run.  

The combination experiments, where 60% vaccination coverage was combined with a 21-day school closure, result in notably later peaks and large decreases in the number of infections. The outbreak peaks five weeks later than in the base run, see Figure 6.   

**Figure:**
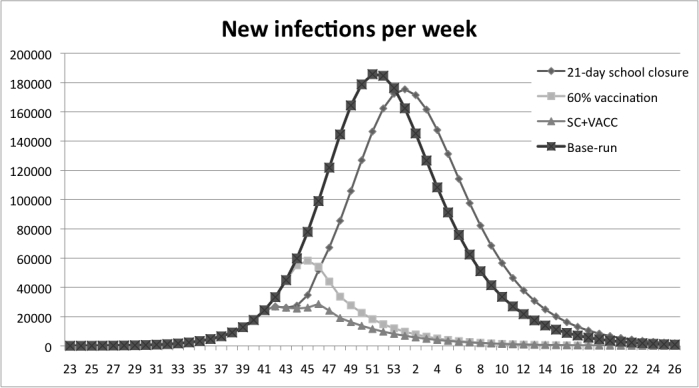

Figure 6: Results from the experiment with a 60% vaccination coverage combined with a 21 day school closure, measured in new infections per week. In the combined scenario the peak of new infections is reached eight weeks earlier than with a 21-day school closure alone and one week later than the scenario with 60% vaccination coverage 

In Figure 7 the combination policy is compared with the school-closure policy. It is shown that with the combination policy the peak is reached earlier than the school-closure policy. The combination strategy is more efficient in delaying the peak than the pure vaccination strategy; see Figure 8 where the peak is delayed one week when the strategies are combined. The overall reduction in number of infections is presented in Table 4. A 21- day school closure decreases the total number of infected by 1% only. The vaccination policy with coverage of 60% decreases the total number of infected by 79%. The combined strategy decreases infections by 86% compared to the base-run. 

**Figure:**
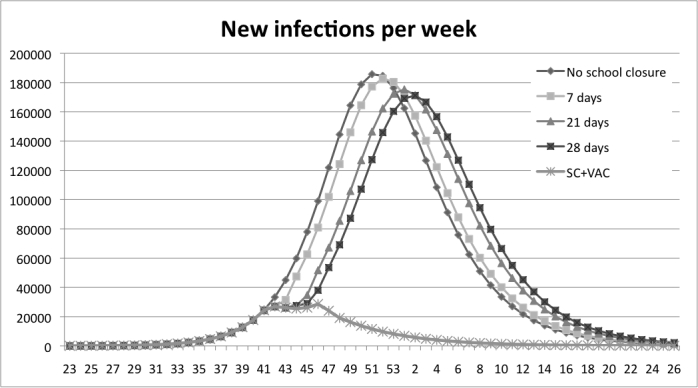

Figure 7: A comparison between the combination policy and the school-closure policy. The peak is reached earlier than the school-closure policy at all different closing times.  

**Figure:**
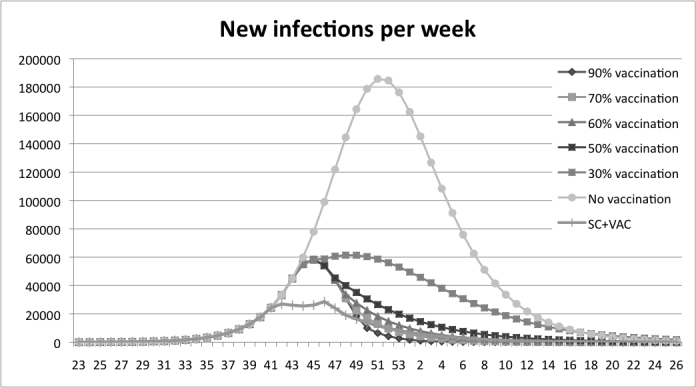

Figure 8: A comparison between the combination policy and the vaccination policy. The combination policy delays the peak compared to all vaccination coverage-levels except for the coverage of 30%.    

**Table:** 

Intervention	Infected	Decrease
No intervention	2588164	0%
21 day school closure	2561902	1%
60% vaccination	555514	79%
Combination: School closure +vaccination	365080	86%

Table 4: Results from the experiments, comparing the decrease in number of infections from the different strategies. The combination strategy produces the largest reduction, 86% compared to the base run with no vaccination. 

## Discussion

The experiments reveal fundamental differences in the effectiveness of a school closure and a vaccination program. The experiment with school closure yields only a small decrease in the total number of infected; it does however delay the spread of infection. Other studies also show that school closure alone is not sufficient to halt the spread of pandemic influenza [11]. Vaccination on the other hand has a large effect on the number of infections; all scenarios reduce the total number of infected by more than 50%. Our results show that the difference in numbers of infected between vaccination coverage of 50% and 90% is relatively small. This behaviour is due to the start time of the vaccination program. An earlier start with fewer individuals already infected would lead to a larger difference between the scenarios with different vaccination coverage. The combination of vaccination and school closure flattens out the peak substantially and reduces the total number of infected by 86% compared to the base-run, which is more than any vaccination coverage alone. A combination of intervention measures is, according to our results, the most effective approach for mitigating the spread of a pandemic influenza. Several parameters regarding the spread of influenza, such as the effect of weather and temperature, social structures and geographical differences in contact structures have been excluded from the model. Studies show that these are all important aspects of how influenza is transmitted [8] [12], but such a level of detail is not relevant for the experiments performed in this study. A simple model of the type here presented should not be considered as a tool for making the best possible forecasts on outbreak sizes and timings. We argue instead that it should rather be viewed as a useful tool for evaluating different policies and parameter values, receiving quick results that are accurate enough to make decisions about whether to investigate the given policy further for instance with another simulation model.

## Funding Information 

This research is supported by The National Board of Health and Welfare (Socialstyrelsen). 

## Competing Interests 

The authors have declared that no competing interests exist. 

## Appendix A

### A.1 Basic structure

Each age group has an identical model structure, which consists of a constant population $(N)$ that is spread out in the following stocks (This is a somewhat simplified description, infected are actually represented by four stocks, one for each disease-profile):

- Susceptible $(S)$
- Latent $(L)$
- Infected $(I)$
- Recovered $(R)$

The value of $S$ is $N-(L+I+R)$. $I$ is initially given an arbitrary value. $L$ and $R$ are initially empty. The stocks are connected by flows representing potential state transitions for individuals in the population, yielding the following structure where → represents a flow between two stocks:

\begin{equation*}S\rightarrow L \rightarrow I \rightarrow R\end{equation*}

 


The following additional constants and variables are used to calculate the

rates of the flows:

- The number of contacts per individual $(c)$
- Infectiousness profile $(\beta)$
- Latency-time constant $(\rho)$
- Recovery-time constant $(\gamma)$
- Infected from other age groups $(i)$

 


The rate equations look as follows:

\begin{equation*}\[ (S\rightarrow L) = S (I+i) \frac{c \beta}{N} \]\end{equation*}

\begin{equation*}(L\rightarrow I) = \frac{L} {\rho}\end{equation*}

\begin{equation*}(I\rightarrow R) = \frac{I} {\gamma}\end{equation*}

### A.2 Implementation of intervention measures

The basic model is extended with two intervention measures, school closure and vaccination.

#### A.2.1 School closure

A school closure leads to a reduction of contacts during the time it is in effect. Contact reduction takes place within the age group 0-19, between 0-19 and the other age groups. The schools close one time only during a simulation when the number of infected passes a certain threshold and lasts for a predetermined number of days. This is implemented in an AnyLogic event-function which is executed cyclically, described here in pseudo-code:


if NOT closedBefore:
     if NOT schoolClosed:
          shareOfInfected = I/N
          if shareOfInfected >= closureThreshold:
               schoolClosed = TRUE 
               startTime = timeStep 
               endTime = startTime + closureLength
               reduceContacts()
     else if schoolClosed AND endTime - timeStep == 0:
          closedBefore = TRUE 
           restoreContacts()
  
            


The reduceContacts and restoreContacts functions update a table containing contact-data.

#### A.2.2 Vaccination program

The vaccination program consists of doses being distributed to the population during the time the vaccination is in effect. Two doses per person are distributed to people aged from 6 months to 13 years; the rest of the population receives a single dose only. Distribution of the second dose starts when the whole share of population supposed to be vaccinated have received their first dose and at least 3 weeks have passed since distribution of the first dose started. 80% of those receiving one dose, 85% of those receiving two doses and 100% of the vaccinated from the age-group 60+ become fully immune after vaccination.  

In order to implement a vaccination program the model has to be extended in several ways. New stocks are added:

- Susceptible receiving vaccination $(S_{v})$
- Susceptible not receiving vaccination $(S_{!v})$
- Susceptible after first dose $(S_{v1})$
- Susceptible after second dose $(S_{v2})$
- Latent after first dose $(L_{v1})$
- Latent after second dose $(L_{v2})$
- Infected after first dose $(I_{v1})$
- Infected after second dose $(I_{v2})$
- Immune $(M)$

Susceptible can become infected, receive vaccination and remain susceptible or become immune:

\begin{equation*}S_{v} \rightarrow L \rightarrow I \rightarrow R\end{equation*},

 

\begin{equation*}\quad S_{v} \rightarrow S_{v1}\end{equation*}

\begin{equation*}\quad S_{v} \rightarrow M\end{equation*}

Susceptible after dose 1 can become infected, receive the second dose and remain susceptible or become immune:

\begin{equation*}S_{v1}\rightarrow L_{v1}\rightarrow I_{v1} \rightarrow R\end{equation*}

\begin{equation*}\quad S_{v1} \rightarrow S_{v2}\end{equation*}

\begin{equation*}\quad S_{v1} \rightarrow M\end{equation*}

Susceptible after dose 2 can become infected:

\begin{equation*}S_{v2} \rightarrow L_{v2}\rightarrow I_{v2} \rightarrow R\end{equation*}

Additional variables are added:

- Doses distributed per day; _first dose $(d_{v1})$
- Doses distributed per day; second dose $(d_{v2})$
- Share of immunity; first dose $(\theta_{v1})$
- Share of immunity; second dose \begin{equation*} $(\theta_{v2})\end{equation*}

 


Initially $d_{v1}$_ _and $d_{v2}$_ _are set to 0, at a given time they are set to a value that represents the number of vaccinations performed per day

The following flow equations determine the rates:

\begin{equation*}(S_{v}\rightarrow L) = S_{v}(I+I_{v1}+I_{v2}+i)\frac{c \beta}{N}\end{equation*}

\begin{equation*}(S_{v1}\rightarrow L_{v1}) = S_{v1}(I+I_{v1}+I_{v2}+i)\frac{c \beta}{N}\end{equation*}

\begin{equation*}(S_{v2}\rightarrow L_{v2}) = S_{v2}(I+I_{v1}+I_{v2}+i)\frac{c \beta}{N}\end{equation*}

\begin{equation*}(S_{v}\rightarrow S_{v1}) = d_{v1}\theta_{v1}\end{equation*}

\begin{equation*}(S_{v}\rightarrow M) = d_{v1}(1-\theta_{v1})\end{equation*}

\begin{equation*}(S_{v1}\rightarrow S_{v2}) = d_{v2}\theta_{v2}\end{equation*}

\begin{equation*}(S_{v1}\rightarrow M) = d_{v2}(1-\theta_{v2})\end{equation*}

## References

[ref-1076971455] Brouwers L, Camitz M, Cakici B, Mäkilä K, Saretok P. 2009. MicroSim: Modeling the Swedish Population. arXiv:0902.0901. Available from: http://arxiv.org/abs/0902.0901.

[ref-3412818448] Forrester, J. W. 1968. Principles of Systems. Cambridge MA: Productivity Press

[ref-2858488146] Sterman JD. 2001. System Dynamics Modeling: Tools for learning in a Complex World. California Management Review. 2001;43(4).

[ref-1518283832] Statistics Sweden (Statistiska centralbyrån, SCB). 2009. Sweden's Population by sex and age on 31/12/2008. Homepage on the Internet: http://www.scb.se/Pages/TableAndChart____262460.aspx (accessed January 20, 2010)

[ref-1182154396] Wallinga J, Teunis P, Kretzschmar M. Using data on social contacts to estimate age-specific transmission parameters for respiratory-spread infectious agents. Am J Epidemiol. 2006 Nov 15;164(10):936-44. Epub 2006 Sep 12. 16968863 10.1093/aje/kwj317

[ref-541665532] Carrat F, Vergu E, Ferguson NM, Lemaitre M, Cauchemez S, Leach S, Valleron AJ. Time lines of infection and disease in human influenza: a review of volunteer challenge studies. Am J Epidemiol. 2008 Apr 1;167(7):775-85. Epub 2008 Jan 29. 18230677 10.1093/aje/kwm375

[ref-2478233995] Fraser C, Donnelly CA, Cauchemez S, Hanage WP, Van Kerkhove MD, Hollingsworth TD, Griffin J, Baggaley RF, Jenkins HE, Lyons EJ, Jombart T, Hinsley WR, Grassly NC, Balloux F, Ghani AC, Ferguson NM, Rambaut A, Pybus OG, Lopez-Gatell H, Alpuche-Aranda CM, Chapela IB, Zavala EP, Guevara DM, Checchi F, Garcia E, Hugonnet S, Roth C; WHO Rapid Pandemic Assessment Collaboration. Pandemic potential of a strain of influenza A (H1N1): early findings. Science. 2009 Jun 19;324(5934):1557-61. Epub 2009 May 11. 19433588 10.1126/science.1176062 PMC3735127

[ref-4104321799] Brouwers L, Cakici B, Camitz M, Tegnell A, Boman M. Economic consequences to society of pandemic H1N1 influenza 2009 - preliminary results for Sweden. Euro Surveill. 2009 Sep 17;14(37). pii: 19333. 19761738 10.2807/ese.14.37.19333-en

[ref-3823302931] Baker MG, Wilson N, Huang QS, Paine S, Lopez L, Bandaranayake D, Tobias M, Mason K, Mackereth GF, Jacobs M, Thornley C, Roberts S, McArthur C. Pandemic influenza A(H1N1)v in New Zealand: the experience from April to August 2009. Euro Surveill. 2009 Aug 27;14(34). pii: 19319. 19712648 10.2807/ese.14.34.19319-en

[ref-437033077] The National Board of Health and Welfare (Socialstyrelsen). 2009. Vaccineringen går enligt plan. [Vaccination is going according to plan]. Homepage on the Internet: http://www.socialstyrelsen.se/pressrum/nyhetsarkiv/vaccineringengarenligtplan. (accessed January 20, 2010)

[ref-1660480514] World Health Organization. 2009. Reducing transmission of pandemic (H1N1) 2009 in school settings. Available from: http://www.who.int/csr/resources/publications/swineflu/reducing_transmission_h1n1_2009/en/index.html

[ref-542295799] Lowen AC, Mubareka S, Steel J, Palese P. Influenza virus transmission is dependent on relative humidity and temperature. PLoS Pathog. 2007 Oct 19;3(10):1470-6. 17953482 10.1371/journal.ppat.0030151 PMC2034399

